# Expert Perspectives on Developing Mixed Reality Software for Benign Paroxysmal Positional Vertigo Management: A Qualitative Study

**DOI:** 10.7759/cureus.111593

**Published:** 2026-06-27

**Authors:** Muhammad Nu'aim Ishak, Muhammad Anas Ismail, Ismahafezi Ismail, Nor Kamaruzaman Esa, Mohd Sayuti Razali, Salman Amiruddin

**Affiliations:** 1 Department of Otorhinolaryngology, Faculty of Medicine, Universiti Sultan Zainal Abidin, Kuala Terengganu, MYS; 2 Department of Otorhinolaryngology, Hospital Sultan Zainal Abidin, Kuala Terengganu, MYS; 3 Centre of Multimedia Studies, Faculty of Informatics and Computing, Universiti Sultan Zainal Abidin, Kuala Terengganu, MYS

**Keywords:** benign paroxysmal positional vertigo, canalith repositioning maneuver, immersive technology, mixed reality, qualitative study, vestibular rehabilitation

## Abstract

Background: Benign paroxysmal positional vertigo (BPPV) is the most common peripheral vestibular disorder, yet its management often varies due to inconsistent maneuver execution and clinician training. Mixed reality offers potential to standardize procedures and enhance user guidance. This study explored expert perspectives to inform the development of mixed reality software designed to facilitate the diagnosis and management of BPPV.

Methods: A qualitative descriptive study was conducted among 17 clinicians with experience in vestibular care. Participants completed a structured online questionnaire containing open-ended items, and responses were analyzed thematically to identify key design priorities and safety considerations.

Results: Thematic analysis identified four major domains: Essential Clinical Features, Functional Enhancements, Perceived Adverse Effects, and Implementation Considerations. Experts highlighted the need for step-by-step procedural guidance, angle-specific head and body measurements, visual and interactive aids, and tools for nystagmus detection. Potential adverse effects, including eye strain, vertigo exacerbation, and nausea, were also emphasized. Implementation priorities included high-definition display quality, lightweight hardware, affordability, and ease of use.

Conclusion: Expert feedback underscored the need for mixed reality systems that prioritize procedural accuracy, usability, and safety. These findings provide an expert-informed framework for a user-centered mixed reality prototype that can be refined and evaluated in future clinical settings.

## Introduction

Vertigo is the false perception of motion in the absence of actual movement and is commonly described as spinning, tilting, or imbalance. It is a frequent clinical complaint, with approximately 15%-20% of adults reporting dizziness annually in population-based studies [1]. Vertigo may arise from central or peripheral causes, of which benign paroxysmal positional vertigo (BPPV) is the most prevalent peripheral vestibular disorder [2]. BPPV results from the displacement of otoconia into the semicircular canals, leading to brief, position-triggered episodes of vertigo. Otoconia are calcium carbonate particles that are normally located in the utricle. Although considered benign, BPPV significantly affects quality of life and is associated with high recurrence rates, ranging from 13.7% to 48% within one year and up to 65% with longer follow-up [3].

The diagnosis and management of BPPV rely primarily on positional maneuvers such as the Dix-Hallpike test and canalith repositioning procedures, including the Epley maneuver [4,5]. Successful canalith repositioning maneuvers restore displaced otoconia to the utricle, which typically results in resolution of vertiginous symptoms. The effectiveness of these maneuvers depends on accurate head and body positioning relative to the affected semicircular canal. However, considerable variability exists in clinical practice, influenced by differences in clinician training, experience, and confidence in performing vestibular maneuvers. Inconsistent execution may lead to diagnostic uncertainty, incomplete symptom resolution, or unnecessary investigations, highlighting the need for approaches that facilitate more precise and reproducible maneuver performance [6].

Recent advances in immersive technologies, including virtual and mixed reality (MR), have generated interest in their applications in medical education, procedural guidance, and rehabilitation [7-9]. Mixed reality differs from fully immersive virtual reality by allowing digital overlays to be integrated into the real-world environment, enabling users to maintain spatial awareness while receiving virtual guidance. This characteristic is particularly relevant to vestibular disorders, where preserving environmental context is important and excessive sensory immersion may exacerbate symptoms.

In the context of BPPV, the clinical rationale for immersive software is closely linked to the gravity-dependent nature of canalith repositioning maneuvers. Accurate head angulation and sequential positioning are essential to guide displaced otoconia through the semicircular canals toward the utricle. When integrated with head-mounted displays incorporating motion and orientation sensing, such systems have the potential to provide real-time visual feedback on head position and movement, thereby facilitating more accurate and reproducible execution of diagnostic and therapeutic maneuvers.

This study aimed to explore expert clinical perspectives on the design requirements, functional priorities, and safety considerations for a mixed reality software prototype intended to support the diagnosis and management of BPPV. Specifically, the study aimed to address three research questions: (1) what clinical features are considered essential for an MR-guided BPPV diagnostic and management system, (2) what functional enhancements and safety concerns do experts anticipate, and (3) what implementation factors are likely to influence clinical adoption.

## Materials and methods

Study design and setting

This study employed a qualitative descriptive design to explore expert perspectives on the development of mixed reality software for the diagnosis and management of benign paroxysmal positional vertigo (BPPV). The study was conducted between June 2024 and December 2024 in Terengganu, Malaysia, across two specific institutions: Hospital Sultanah Nur Zahirah and Hospital Sultan Zainal Abidin. These two hospitals were purposefully selected because they are the main tertiary referral centers in the region and are equipped with dedicated otorhinolaryngology (ORL) specialist departments capable of managing complex vestibular disorders.

Participants and sampling strategy

Purposive sampling was utilized to recruit clinicians with direct, sustained experience in BPPV management, ensuring domain expertise relevant to software design. Inclusion criteria required at least five years of clinical experience in vestibular care and English proficiency. Potential participants were identified and approached through the respective heads of department at each institution, who facilitated initial contact based on clinical role, departmental affiliation, and years of experience in vestibular care; interested clinicians were then invited directly by the research team to complete the structured questionnaire.

To accurately reflect the local clinical care pathway, recruitment heavily prioritized otorhinolaryngologists, as they serve as the primary specialists diagnosing and performing canalith repositioning maneuvers within these regional referral centers. Emergency physicians were recruited as first-contact clinicians who frequently manage acute BPPV presentations before referral to ORL, while physiotherapists were included as practitioners who perform repositioning maneuvers during post-diagnostic vestibular rehabilitation in outpatient and community contexts. Together, the three clinician groups capture the full spectrum of BPPV diagnostic and management contexts relevant to the design of an Extended Reality guidance system.

Ultimately, 17 clinicians participated in the study. The composition was predominantly weighted toward otorhinolaryngologists, reflecting a constraint of the clinical environment in which BPPV management at these centers is concentrated within the otorhinolaryngology specialty; only a small number of emergency physicians and physiotherapists regularly perform or supervise the relevant procedures to the threshold of clinical experience required for inclusion. The exact composition of the final sample is reported in the Results section (Participant Demographics).

Data collection instrument and procedure

Data were collected using a structured online questionnaire administered via Google Forms. The questionnaire was developed de novo by the research team, as no validated instrument currently exists for this specific domain, given the emerging nature of this topic. It was grounded in established clinical principles for benign paroxysmal positional vertigo management and in the published literature on immersive technologies in healthcare [7,10]. The questionnaire comprised four main domains: participant demographics; current practices and challenges in performing the Dix-Hallpike test and canalith repositioning maneuvers; preferred features of the immersive software; and anticipated adverse effects.

Prior to distribution, the questionnaire underwent expert content validation by a senior otorhinolaryngologist who reviewed the instrument for clinical relevance and face validity. The instrument was deliberately structured to capture rich, in-depth qualitative data through open-ended questions with no character limit. Furthermore, participants were guided by specific, probing sub-questions designed to elicit comprehensive narrative insights regarding their daily clinical workflows and firsthand experiences. The full questionnaire is available in Appendices 1 and 2.

Conceptual framework

Expert feedback from the 17 clinician participants was used to define the functional and clinical specifications that formed the conceptual basis for the mixed reality (MR) software prototype. These specifications informed the initial design framework, including core functionalities, safety considerations, and usability requirements. The subsequent development phase will involve iterative user experience testing and further expert evaluation before progression to formal clinical validation.

Data analysis and thematic coding

Data were analyzed using a hybrid framework analysis approach, which integrated deductive categorization based on the study's predefined domains with inductive thematic coding to capture emergent expert insights. The analysis followed the six-phase framework described by Braun and Clarke and was managed using Microsoft Excel (Microsoft Corporation, Redmond, Washington, USA) to maintain a systematic coding matrix [11].

The final sample size was not predetermined but rather dictated by thematic saturation, which was continuously assessed using a constant comparative method, with responses evaluated in consecutive batches of five. Saturation was assessed holistically across all three professional groups rather than independently within each group, a decision supported by the procedural standardization of BPPV management approaches across clinical disciplines.

To ensure rigor, two researchers independently reviewed and coded the raw responses. Inter-coder reliability was established through negotiated agreement, consistent with the qualitative paradigm adopted in this study and a widely accepted approach in reflexive thematic analysis. Discrepancies between the researchers were resolved through iterative discussion until consensus was achieved.

To illustrate the coding process, the response "specific angle of head movement for Dix-Hallpike and Epley maneuver" was coded as an angle-specific measurement and assigned to the theme Essential Clinical Features. The response "neck and back headache" was coded as musculoskeletal discomfort and assigned to the theme Perceived Adverse Effects. The response "suitable for person wearing glasses" was coded as compatibility with eyewear and assigned to the theme Implementation Considerations.

Code stability was achieved as the analysis approached two-thirds of the participant cohort, after which subsequent responses confirmed and enriched established concepts rather than introducing new thematic categories. This iterative monitoring of data adequacy ensured that the sample size was sufficient to capture a comprehensive range of expert perspectives. Peer debriefing with a senior reviewer was also conducted to audit the final codebook and ensure analytic consistency.

A keyword-based clustering function within standard qualitative data analysis software assisted with the initial organization of raw responses into provisional groupings. All final coding decisions, theme development, and interpretations were conducted manually by the two researchers. This manual oversight ensured that the findings remained grounded in the clinical context and independent of potential algorithmic bias.

## Results

Participant demographics

A total of 17 experts participated in the study. The demographic distribution consisted of 13 otorhinolaryngologists (76.5%), three emergency physicians (17.6%), and one physiotherapist (5.9%). Regarding clinical experience, all participants (100%) possessed more than five years of specialized practice in diagnosing and managing BPPV and related vestibular disorders. The following sections summarize the findings across the four primary domains, with detailed subthemes, representative quotes, and design implications provided in Table 1. While all 17 participants provided narrative feedback and agreed on the general need for standardized, safe, and user-friendly BPPV management tools, the frequency counts (n) detailed below indicate the specific number of experts who explicitly suggested a granular feature without prompting. A complete summary of the raw narrative responses for all domains is available in Appendices 1 and 2.

**Table 1:** Thematic domains, subthemes, and expert-identified design priorities for the mixed reality BPPV software. The table summarizes the four thematic domains and associated subthemes identified from expert qualitative feedback. Frequency values (n) indicate the number of participants who mentioned each subtheme.

Theme	Key subthemes	Frequency of mention (n)	Representative quote	Design implication
Essential Clinical Features	Step-by-step guidance	3	"Specific angle of head movement for Dix-Hallpike and Epley maneuver."	Standardize maneuver execution to reduce clinical variability.
	Diagnostic analyzer	2		
	Angle-specific measurements	2		
Functional Enhancements	Visual aids	4	"Visual aids showing the movement of otoliths during maneuvers."	Enhance clinician training and patient education through visualization.
	Interactive elements	3		
	Nystagmus detection	3		
	Multilingual support	3		
Perceived Adverse Effects	Eye strain	6	"Could induce more vertiginous effect for first-time/unfamiliar users."	Implement adjustable display settings and short session durations.
	Vertigo exacerbation	6		
	Nausea and vomiting	3		
Implementation Considerations	HD display quality	8	"Incorporate clinician mode for real-time monitoring and control during sessions."	Prioritize high-performance, portable hardware for sustainable clinical use.
	Lightweight design	3		
	Affordability	2		
	Clinician mode	1		

Comparison across professional groups

A descriptive comparison of coded responses across professional groups revealed a notable difference in thematic emphasis. Otorhinolaryngologists contributed proportionally more codes relating to Implementation Considerations and Perceived Adverse Effects, reflecting their greater procedural familiarity with the biomechanical and clinical nuances of BPPV management. In contrast, emergency physicians and physiotherapists contributed proportionally more codes relating to Essential Clinical Features, consistent with their comparatively lower frequency of independently performing these procedures. This difference was one of emphasis and depth rather than disagreement, as no contradictory requirements emerged between groups.

Essential clinical features

This domain encompasses elements necessary to ensure the software effectively supports diagnostic and therapeutic maneuvers for BPPV. Participants emphasized the importance of step-by-step procedural guidance (n=3) and adherence to standard protocols such as the Dix-Hallpike and Epley maneuvers. Additional features included angle-specific head and body measurements (n=2), real-time positional feedback (n=1), and progress-tracking tools (n=1). These priorities confirm a strong clinical demand for tools that ensure procedural accuracy and reproducibility, underscoring the perceived value of tool-assisted guidance during canalith repositioning maneuvers.

Functional enhancements

This domain focused on improving usability and engagement. Commonly suggested features included visual aids (n=4), interactive elements (n=3), and nystagmus detection tools (n=3). Other valued functions were multilingual support (n=3), eye-fixation pointers (n=2), and simulation videos (n=2). Participants also proposed voice-guided instruction, haptic feedback, and eye-tracking integration to support both training and clinical application. These inputs suggest that clinicians view the platform not only as a diagnostic aid but also as a flexible educational and rehabilitation tool adaptable to diverse clinical settings.

Perceived adverse effects

This domain reflected concerns related to user safety and tolerance. The most frequently cited issues were eye strain (n=6), vertigo exacerbation (n=6), and nausea or vomiting (n=3), particularly among first-time users or individuals with heightened sensory sensitivity. Less frequently reported concerns included headache, neck or back discomfort, claustrophobia, and risk of falls. These responses indicate the importance of ergonomic design, visual comfort, and adjustable display parameters to minimize adverse effects during use.

Implementation considerations

This domain addressed practical aspects of real-world deployment. A high-definition display (n=8) was the most commonly requested feature, followed by lightweight design (n=3), affordability (n=2), and ease of use (n=2). Additional recommendations included adjustable headgear, Frenzel goggle integration, and clinician-mode controls. Together, these considerations reflect clinicians’ awareness of feasibility constraints and the need for solutions that balance technical precision with usability, comfort, and cost-effectiveness to support sustainable integration into clinical workflows.

## Discussion

This qualitative study explored expert perspectives to inform the early development of mixed reality software for the diagnosis and management of benign paroxysmal positional vertigo. Four thematic domains were identified, reflecting clinical priorities related to procedural accuracy, usability, safety, and implementation feasibility. By incorporating multidisciplinary expert input at the pre-development stage, the findings provide a clinically grounded framework for guiding user-centered software design.

The domain of Essential Clinical Features highlights the central importance of procedural accuracy in BPPV diagnosis and treatment. Rather than focusing on specific features alone, expert feedback reflects broader concern regarding variability in maneuver execution and clinician confidence. This aligns with existing evidence demonstrating substantial inconsistency in head positioning during canalith repositioning maneuvers and supports the rationale, as perceived by experts, for immersive systems that could provide real-time positional guidance to enhance standardization and reproducibility [6].

Functional Enhancements focused on features intended to improve usability, engagement, and interpretability. Visual aids, interactive elements, and nystagmus detection tools were frequently suggested, alongside options for multilingual support and simulation-based learning. These findings are consistent with previous studies reporting that interactive and visually guided technologies can enhance user understanding and satisfaction in vestibular rehabilitation contexts [12,13]. Experts clearly conceptualized the software not only as a diagnostic aid but also as a potential educational resource for training and skill reinforcement across different clinical environments.

Safety considerations emerged as a key theme within the Perceived Adverse Effects domain. Concerns regarding eye strain, vertigo exacerbation, and nausea reflect well-recognized challenges associated with immersive environments, particularly among vestibular-sensitive individuals. These findings are consistent with reports of simulator-related discomfort in virtual and augmented reality applications and highlight the importance of ergonomic design, adjustable visual parameters, and clinician supervision to ensure safe implementation [10,14].

The Implementation Considerations domain highlighted practical factors influencing feasibility and adoption. Experts prioritized high-definition displays, lightweight hardware, affordability, and ease of use, reflecting awareness of both clinical workflow constraints and resource limitations. Additional suggestions, such as adjustable headgear and clinician-mode controls, further emphasized the need for adaptability and comfort. These considerations reinforce the view that technical capability alone is insufficient for successful integration, and that usability and cost-effectiveness are critical determinants of sustained clinical uptake [15].

Although the initial development plan considered a fully immersive virtual reality (VR) approach, early expert feedback raised concerns that such immersion could potentially worsen symptoms in vestibular-sensitive individuals and increase the risk of adverse effects. In response, the design focus shifted toward a mixed reality (MR) model aimed at mitigating these risks. By preserving visual access to the physical environment while providing virtual guidance, MR was perceived as a more tolerable and safer option for vestibular assessment and training.

A key strength of this study is the early integration of multidisciplinary expert input and the use of manual thematic analysis to guide software development. Applying a user-centered design approach at the pre-development stage supports closer alignment with clinical practice and promotes end-user engagement. The inclusion of clinicians from otorhinolaryngology, emergency medicine, and physiotherapy enhances the breadth and robustness of the findings.

Several limitations should be acknowledged. The sample size, while consistent with thematic saturation and the limited availability of eligible experts, remains relatively small. The sample was also heavily skewed toward otorhinolaryngologists, as this specialty is primarily responsible for diagnosing and managing BPPV. Emergency physicians and physiotherapists were included because they also encounter and manage BPPV in their respective practice settings, though in smaller numbers. Data were collected through a structured online questionnaire rather than semi-structured interviews. This format likely limited the depth and nuance of narrative responses. The study was also restricted to two regional centers, which may limit the transferability of findings to settings with different referral pathways or resource availability. 

The findings will inform subsequent phases of prototype refinement, including usability testing, expert evaluation, and clinical performance assessment. Future work will extend to patient and non-specialist use, with the objective of delivering a clinically grounded, user-centered tool to support accurate BPPV diagnosis and management. More broadly, these qualitative insights provide an expert-informed framework that other researchers or developers can use as a foundation for designing immersive software that supports clinical maneuvers.

## Conclusions

This study provides expert-informed guidance for the development of mixed reality software to support the diagnosis and management of benign paroxysmal positional vertigo. Key priorities identified include procedural accuracy, user engagement, safety, and practical usability. These findings offer a clinically grounded framework to guide the design of a user-centered mixed reality prototype, which will be further refined and evaluated in subsequent phases of development.

## Figures and Tables

**Figure 1:**
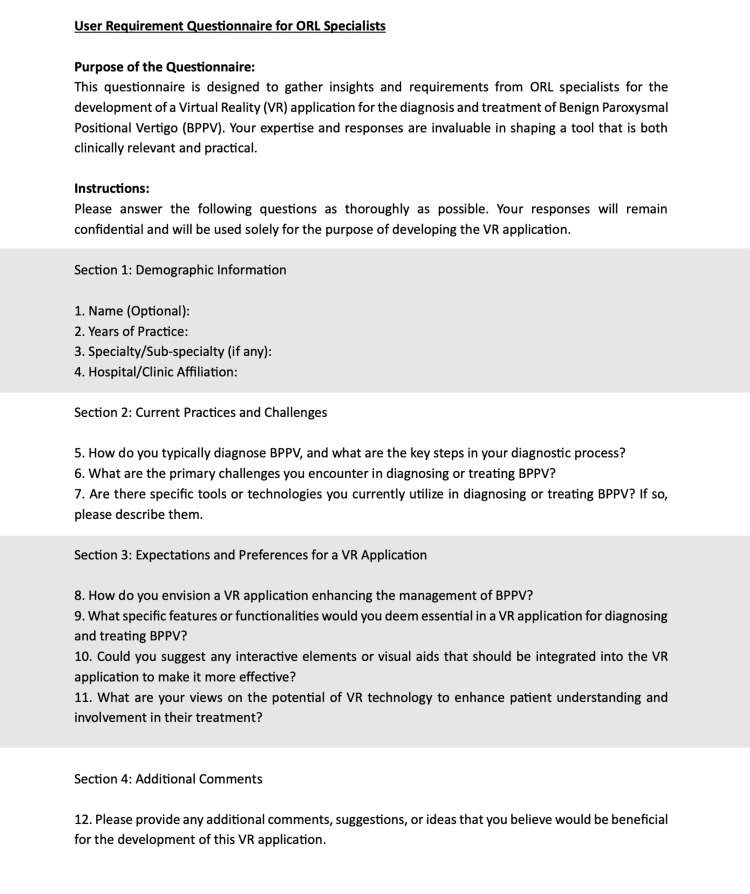
Questionnaire. ORL: otorhinolaryngology.

## Appendices

Appendix 1 

Appendix 2 

**Table 2:** Participant responses with descriptive summary.

Primary domain	Responses	n	Descriptive summary of participant responses
Essential Clinical Features	Analyzer	2	Participants highlighted the necessity for the software to provide clear, step-by-step guidance for standard BPPV treatments, such as the Dix-Hallpike and Epley maneuvers. There was a specific emphasis on the system's ability to measure the precise angle of head movement required during these procedures. Furthermore, experts requested real-time feedback on head and body positioning, as well as tools to track patient progress over time.
	Step-by-step guidance	3	
	Follow BPPV treatment protocol	1	
	Proper technique guidance	1	
	Angle-specific measurement	2	
	Real-time feedback on head/body positioning	1	
	Progress tracking	1	
Functional Enhancements	Visual aids	4	To enhance usability, participants frequently recommended integrating visual aids, including interactive tutorials and simulation videos that show the movement of otoliths during maneuvers. Tools for better visualization of subtle nystagmus, including eye tracking and nystagmograph integration, were strongly requested. Other functional additions included multilingual support, eye fixation pointers, clear voice-guided audio instructions, and sensory features like calming music or haptic feedback.
	Interactive features	3	
	Nystagmus detection device	3	
	Multilingual support	3	
	Eye fixation pointer	2	
	Music	2	
	Simulation video	2	
	Eye tracking	1	
	Noise cancelling	1	
	Voice-guided instruction	1	
	360-degree view	1	
	Mixed reality	1	
	Haptic feedback	1	
	Clear instructional guidance	1	
	Otoconia movement visualization	1	
	Safety warnings	1	
Perceived Adverse Effects	Eye strain	6	Safety concerns primarily revolved around the potential for the immersive software to cause severe eye strain or directly induce/exacerbate the patient's vertigo and dizziness. Participants also noted a significant risk of triggering nausea, vomiting, or motion sickness. Additional concerns included neck and back discomfort (headaches), claustrophobia, and the risk of the patient falling due to an inability to view their physical surroundings.
	Vertigo	6	
	Nausea and vomiting	3	
	Jeopardize clinical judgement	1	
	Neck and back pain	1	
	Headache	1	
	Claustrophobia	1	
	Risk of fall	1	
	Discomfort in older patients	1	
Implementation Considerations	High-definition screen	8	For practical clinical use, the most prominent requirement was a high-resolution, high-definition display for clear visual instruction. Participants stressed that the hardware must be lightweight, user-friendly, and affordable. Practical hardware and design suggestions included comfortable, adjustable head straps, compatibility with prescription glasses or Frenzel goggles, wireless designs (fewer wires attached to the patient), and a "clinician mode" that allows the instructor to monitor and control the session in real-time.
	Light device	3	
	Affordable	2	
	Straps	2	
	Frenzel glass integration	2	
	Easy to use	2	
	Ensure compatibility with a range of VR devices	1	
	Safety gears	1	
	Suitable for person wear glasses	1	
	Safety personnel available	1	
	Incorporate clinician mode for real-time monitoring and control during sessions	1	
	Short duration of assessment	1	
	Comfortable to wear	1	
	Anti-claustrophobic	1	
	Reduced movement needed during the application usage	1	
	Spatial collaboration	1	
	Less wire attach to patient	1	
